# Digital harassment and its mental health impact among healthcare professionals: A scoping review

**DOI:** 10.1002/pcn5.70362

**Published:** 2026-06-11

**Authors:** Kyohei Otani, Tomohiro Kinoshita, Ryota Shindo, Shogo Kurushima

**Affiliations:** ^1^ Department of Psychiatry Kakogawa Central City Hospital Kakogawa Hyogo Japan

**Keywords:** cyberbullying, digital harassment, health personnel, healthcare professionals, mental health, occupational health, occupational stress, scoping review, workplace violence

## Abstract

Digital harassment, including workplace cyberbullying, online defamation, and social media attacks, has been reported among healthcare professionals. This scoping review systematically maps existing evidence on digital harassment and its mental health consequences among healthcare professionals. Following Preferred Reporting Items for Systematic reviews and Meta‐Analyses extension for Scoping Reviews (PRISMA‐ScR) guidelines, we searched MEDLINE, Google Scholar, and Ichushi‐Web through December 2025. Twenty‐four studies from 12 countries were included. Workplace cyberbullying prevalence ranged from 1.5% to 46.6%. Across studies, digital harassment was reported in association with depression, anxiety, burnout, post‐traumatic stress disorder (PTSD) symptoms, and moral injury‐related experiences, although causal relationships could not be established. Seven included studies (28%) addressed pandemic‐related digital harassment, including online abuse and stigmatization of healthcare workers during the COVID‐19 period. Within the English‐ and Japanese‐language literature identified in this review, Japanese studies were limited (three studies). From a consultation‐liaison psychiatry perspective, harassment following ethically mandated actions may relate to moral injury‐related experiences and may warrant further attention to institutional support. Evidence for effective interventions remains scarce. This review suggests that digital harassment may represent an emerging occupational concern with potential mental health implications. The evidence base was predominantly cross‐sectional and methodologically heterogeneous, and findings should therefore be interpreted cautiously. Important needs include institutional policies, legal protections, psychological support systems, and further research on intervention effectiveness and cross‐cultural patterns.

## Graphical abstract

Digital harassment—including workplace cyberbullying, online defamation, and coordinated social media attacks—has been reported among healthcare professionals and may represent an emerging occupational concern. This scoping review of 24 studies from 12 countries found that digital harassment was reported in association with adverse mental health outcomes, including depression, anxiety, burnout, PTSD symptoms, and moral injury–related experiences. Seven included studies addressed COVID‐19–related harassment. The findings suggest that further attention to institutional support, legal consultation pathways, and psychological support systems may be warranted.

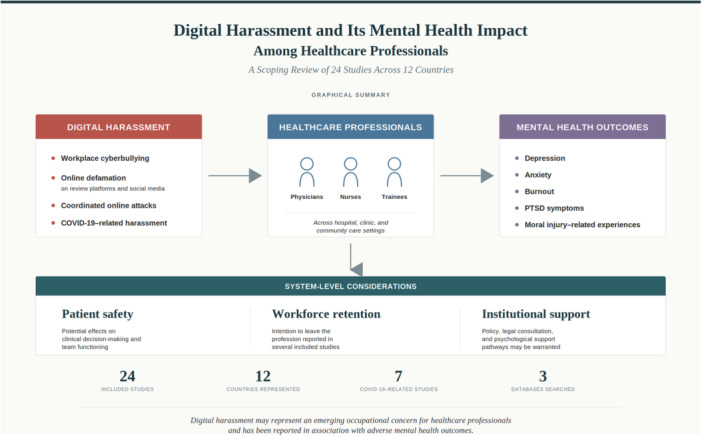

## INTRODUCTION

The rapid expansion of digital communication technologies has substantially changed healthcare delivery, professional communication, and the relationship between healthcare professionals and the public. Electronic health records, telemedicine platforms, and social media have enhanced care coordination and knowledge sharing. However, these technologies have created new avenues for harassment directed at healthcare professionals, which may constitute an emerging occupational concern.^1^, ^2^ Digital harassment encompasses workplace cyberbullying involving hostile communications from colleagues via email or messaging platforms,^3^ online defamation through physician rating websites or social media,^4^ and coordinated social media attacks. Unlike traditional face‐to‐face bullying, digital harassment transcends temporal and spatial boundaries, can persist indefinitely in archived content, and can reach vast audiences instantaneously. Healthcare professionals face particular vulnerability for several reasons. First, healthcare work involves high‐stakes decision‐making under uncertainty and time pressure. When outcomes are unfavorable, patients and families might attribute these to professional incompetence rather than disease severity or medical limitations, expressing dissatisfaction through defamatory reviews or social media posts. Second, healthcare professionals occupy public‐facing roles requiring increasing digital visibility. Many organizations encourage professional social media presence for patient engagement or health promotion. This visibility exposes healthcare workers to public scrutiny and potential targeting by those who disagree with clinical decisions or public health recommendations. Third, healthcare professionals face ethical and legal obligations that can provoke backlash. Mandatory reporting requirements for child abuse or elder abuse may anger those reported, who may retaliate through online defamation.^4^ Implementation of controversial evidence‐based practices—such as vaccination advocacy or provision of reproductive healthcare—can trigger coordinated harassment campaigns. Public health measures, particularly during outbreaks, may require the enforcement of restrictive policies generating public resentment. Fourth, hierarchical healthcare structures create power imbalances facilitating workplace cyberbullying. Trainees and early‐career professionals tend to hesitate to report harassment from supervisors due to fears of retaliation or career consequences. Digital harassment offers perpetrators apparent anonymity while creating permanent records that victims cannot easily escape.^5^ Studies have reported that the COVID‐19 pandemic may have been associated with intensified digital harassment of healthcare professionals.^6^, ^7^ Healthcare workers became symbols of pandemic disruptions, facing online abuse related to infection control measures, presumed responsibility for disease transmission, and alignment with unpopular public health policies. Social media platforms have been reported to amplify these dynamics by facilitating misinformation spread and the coordination of harassment campaigns. The pandemic appears to have exposed inadequacies in institutional protections for healthcare professionals facing digital harassment.^8^, ^9^ The potential mental health consequences appear notable. Unlike traditional workplace bullying occurring within defined contexts, digital harassment creates chronic, unpredictable stress. Victims can encounter harassing content repeatedly across platforms, experience anxiety about who has viewed defamatory material, and face realistic concerns about lasting reputational damage. The public nature of social media harassment may intensify shame and professional identity threats, suggesting that digital harassment may create recurrent and unpredictable stressors that warrant attention within occupational mental health frameworks. Despite increasing attention to this issue, systematic synthesis of evidence specific to healthcare professionals remains limited. Existing reviews have focused on workplace bullying generally, cyberbullying in other populations, or specific contexts such as physician rating websites. No prior scoping review has systematically mapped evidence on digital harassment across diverse healthcare professional populations and contexts. This scoping review therefore aims to (1) systematically identify and characterize studies examining digital harassment of healthcare professionals; (2) describe types, contexts, and prevalence of digital harassment across different settings and professional groups; (3) synthesize evidence on associations between digital harassment and mental health outcomes; (4) identify geographic and methodological gaps; and (5) propose directions for future research and practice.

## METHODS

### Study design and framework

This scoping review followed the Arksey and O'Malley^10^ framework, refined by the Joanna Briggs Institute (JBI),^11^ and is reported according to Preferred Reporting Items for Systematic reviews and Meta‐Analyses extension for Scoping Reviews (PRISMA‐ScR) guidelines^12^ (see Table S2 for the PRISMA‐ScR checklist). This scoping review was not prospectively registered.

### Search strategy

A comprehensive search was conducted in December 2025 across MEDLINE (via PubMed), Google Scholar, and Ichushi‐Web. Search strategies combined three concept groups: (1) healthcare professionals (e.g., “healthcare worker,” “physician,” and “nurse”); (2) digital harassment (e.g., “cyberbullying,” “online harassment,” and “social media attack”); and (3) mental health outcomes (e.g., “mental health,” “burnout,” “post‐traumatic stress disorder [PTSD],” and “moral injury”).

MEDLINE (PubMed) searches used the three‐concept strategy as a free‐text keyword string entered in PubMed's default search interface. Google Scholar was searched as an additional source of gray and supplementary literature. Because of the large number of retrieved records (approximately 17,800), screening was pragmatically limited to the first 200 relevance‐sorted results, from which 74 records were identified as potentially relevant; this approach is consistent with established practice in scoping reviews using non‐indexed databases, whose search results and rankings may vary over time.^10^, ^11^ Digital harassment was operationally defined based on the terminology and definitions used in each included study. Ichushi‐Web searches employed Japanese terms related to healthcare professionals, online defamation or harassment, social media or online reviews, and mental health outcomes. The full search strategy for MEDLINE is provided in Table S1.

No start date restrictions were applied to the search strategy. Searches covered records from database inception to December 31, 2025. The original MEDLINE search identified 156 records, which were combined with the records retrieved from Google Scholar and Ichushi‐Web to form the pooled set of records carried forward to screening and study selection. The MEDLINE/PubMed search strategy used for this original screening is detailed in Table S1; this strategy was clarified during revision to reflect the search as conducted, which did not change the screening process, PRISMA flow, included studies, or conclusions. A Japanese database (Ichushi‐Web) was also searched to capture domestic Japanese‐language literature that may not be indexed in MEDLINE. The full electronic search strategy for MEDLINE is provided in Table S1.

### Eligibility criteria

Studies were included if they (1) examined healthcare professionals as the primary population; (2) addressed digital harassment including workplace cyberbullying, online defamation, or social media attacks; (3) reported mental health outcomes such as depression, anxiety, burnout, PTSD, or turnover intention; and (4) were published in English or Japanese.

All study designs were eligible for inclusion, consistent with the broad mapping purpose of a scoping review. This included quantitative, qualitative, and mixed‐methods studies, as well as commentaries, frameworks, and case reports that directly addressed digital harassment of healthcare professionals, consistent with scoping review methodology. Studies were excluded if they (1) focused exclusively on traditional face‐to‐face bullying; (2) did not report mental health outcomes; (3) examined non‐healthcare populations without separate healthcare subgroup reporting; or (4) were editorials or opinion pieces without specific relevance to digital harassment or mental health in healthcare settings. To address terminological inconsistencies across studies, key definitions and related concepts of digital harassment identified in the literature are summarized in Table 1.

**Table 1:** Characteristics of included studies on digital harassment among healthcare professionals (*N* = 24).

Author, year	Country	Study design	Sample (profession, *n*)	Type of digital harassment	Mental health outcomes	Key findings
Otani et al., 2025^4^	Japan	Case report	Pediatric healthcare professionals	Online defamation via Google Reviews following mandatory reporting	Psychological distress, moral injury, stress	Mandatory reporting triggered digital harassment leading to marked psychological distress; institutional protection is crucial
Farley et al., 2015^3^	United Kingdom	Cross‐sectional survey	Trainee doctors (*n* = 158)	Workplace cyberbullying	Mental strain, job dissatisfaction	46.2% experienced cyberbullying; associated with higher mental strain and lower job satisfaction
La Regina et al., 2021^1^	Italy	Exploratory study	Healthcare providers	Social media aggression	Emotional distress, professional well‐being	Online aggression negatively affected emotional and professional well‐being
Park and Choi, 2019^13^	South Korea	Cross‐sectional survey	Nurses (*n* = 249)	Workplace cyberbullying	Symptom burden, turnover intention	Cyberbullying associated with symptom experience and intention to leave
Ikeda et al., 2022^14^	Japan	Cross‐sectional survey	Healthcare workers	COVID‐19–related cyberbullying	Psychological distress	Pandemic‐related cyberbullying significantly associated with distress
Zhang et al., 2025^15^	International	Systematic review and meta‐analysis	Healthcare workers (21 studies)	Workplace cyberbullying	Various mental health outcomes	Prevalence ranged from 1.5% to 46.6%; nurses particularly affected
Muhonen et al., 2017^16^	Sweden	Cross‐sectional survey	Working adults (*n* = 3371)	Cyberbullying behavior	Well‐being, work engagement	Organizational climate mediated impact of cyberbullying
Choi and Park, 2019^17^	South Korea	Cross‐sectional survey	Nurses	Face‐to‐face and cyberbullying	Burnout, job satisfaction	Organizational culture influenced both bullying types
Kowalski et al., 2018^2^	United States	Cross‐sectional survey	Working adults (*n* = 321)	Cyberbullying, cyberincivility	Psychological distress	All forms associated with adverse mental health
Devi, 2020^6^	Global	Commentary	Healthcare workers	COVID‐19–related online violence	Mental health concerns	Pandemic intensified violence and harassment against HCWs
Rodríguez‐Bolaños et al., 2020^7^	Mexico	Commentary	Healthcare workers	COVID‐19–related harassment	Stress, burnout	Highlighted urgent need for protective measures
Saragih et al., 2022^8^	Global	Systematic review and meta‐analysis	Healthcare workers	Stigmatization, online violence	Anxiety, depression, distress	High prevalence of violence and stigma during COVID‐19
Janoušková et al., 2024^9^	International	Cross‐sectional survey	Healthcare workers	Stigma, discrimination, online harassment	Anxiety, depression, PTSD	Harassment significantly associated with mental health symptoms
Asaoka et al., 2021^18^	Japan	Cross‐sectional survey	Healthcare professionals (*n* = 1421)	Workplace bullying incl. digital forms	Psychological distress	Bullying and patient aggression linked to distress
Chen et al., 2022^19^	China	Cross‐sectional survey	Healthcare workers	Emotional abuse, online violence	Burnout	Workplace violence associated with burnout
Askew et al., 2012^20^	Australia	e‐Cohort study	Doctors (*n* = 747)	Workplace bullying (incl. digital)	Mental health, sick leave	Bullying associated with poorer mental health
Bunce et al., 2024^21^	United Kingdom	Population survey	Employees (general)	Workplace bullying, harassment	Depression, anxiety	Bullying strongly associated with mental disorders
Gillen et al., 2017^22^	International	Cochrane review	Workplace interventions	Bullying prevention	Various	Limited evidence for effective interventions
D'Cruz and Noronha, 2013^5^	International	Conceptual analysis	Employees	Workplace cyberbullying	Psychological impact	Highlighted extended reach of cyberbullying
Coyne et al., 2017^23^	International	Cross‐sectional survey	Employees	Workplace cyberbullying	Mental strain, job satisfaction	Cyberbullying reduced job satisfaction
Kim et al., 2021^24^	Malaysia	Study protocol	TCM practitioners (planned *n* = 1023)	Workplace cyberbullying	Psychological well‐being	Protocol for intervention‐oriented research
Regehr et al., 2023^25^	Canada	Qualitative study	Public health professionals	Online harassment, threats	Psychological distress	Qualitative evidence of severe online abuse during COVID‐19
Cain et al., 2019^26^	United States	Commentary/framework	Medical faculty	Coordinated online attacks	Professional stress	Proposed institutional response framework
Tokeshi et al., 2025^27^	Brazil	Longitudinal study	Medical residents (*n* = 218)	Moral harassment	Anxiety, depression, burnout	>90% reported harassment; mental health worsened over time

*Note*: Definitions of digital harassment varied across studies; therefore, categorization was based on authors' descriptions in each original study.

Abbreviations: HCW, healthcare worker; PTSD, post‐traumatic stress disorder; TCM, traditional Chinese medicine.

### Study selection and data extraction

Search results were imported into reference management software and duplicates removed. Two reviewers (K.O., T.K.) independently screened titles and abstracts. Disagreements were resolved through discussion with a third reviewer (R.S.) when needed. Full‐text articles were retrieved for potentially eligible records.

Two reviewers (K.O., S.K.) independently assessed full‐text articles, documenting exclusion reasons. Inter‐rater reliability was substantial (Cohen's *κ* = 0.86), calculated for the full‐text assessment stage, indicating a high level of inter‐rater reliability. Disagreements were resolved through discussion.

Data extraction employed a standardized form pilot‐tested on five studies. Extracted data included: author and year; study design; country; healthcare professional population; type of digital harassment; mental health outcomes and instruments; key findings including prevalence estimates and effect sizes; and limitations.

For quantitative studies, data included sample characteristics, prevalence estimates with confidence intervals, and statistical associations (correlation coefficients, odds ratios, and regression coefficients). For qualitative studies, key themes were extracted. For systematic reviews, the number of included studies and main conclusions were extracted.

### Data synthesis

Given heterogeneity in designs, definitions, and measures, meta‐analysis was inappropriate. Data were synthesized narratively around: (1) study characteristics and geographic distribution; (2) types and contexts of harassment; (3) mental health outcomes; and (4) research gaps. This approach is consistent with scoping review methodology, which maps evidence breadth rather than generating pooled estimates. As this was a scoping review conducted in accordance with JBI methodological guidance, formal critical appraisal of included studies was not conducted. Methodological characteristics of included studies (study design, sample size, validated measures, and key limitations) are described narratively in the Results, and methodological heterogeneity is acknowledged in the Limitations. Studies explicitly conducted during the COVID‐19 pandemic or directly addressing pandemic‐related harassment were classified as pandemic‐related studies.

## RESULTS

### Study selection

The search identified 245 records: MEDLINE (*n* = 156), Google Scholar (*n* = 74), and Ichushi‐Web (*n* = 15). After removing 65 duplicates, 180 unique records underwent title and abstract screening, excluding 120 records: traditional bullying only (*n* = 48), non‐healthcare population (*n* = 32), no mental health outcomes (*n* = 24), and other reasons (*n* = 16). Non‐healthcare populations were excluded at both title/abstract and full‐text stages based on predefined eligibility criteria.

**Figure 1:**
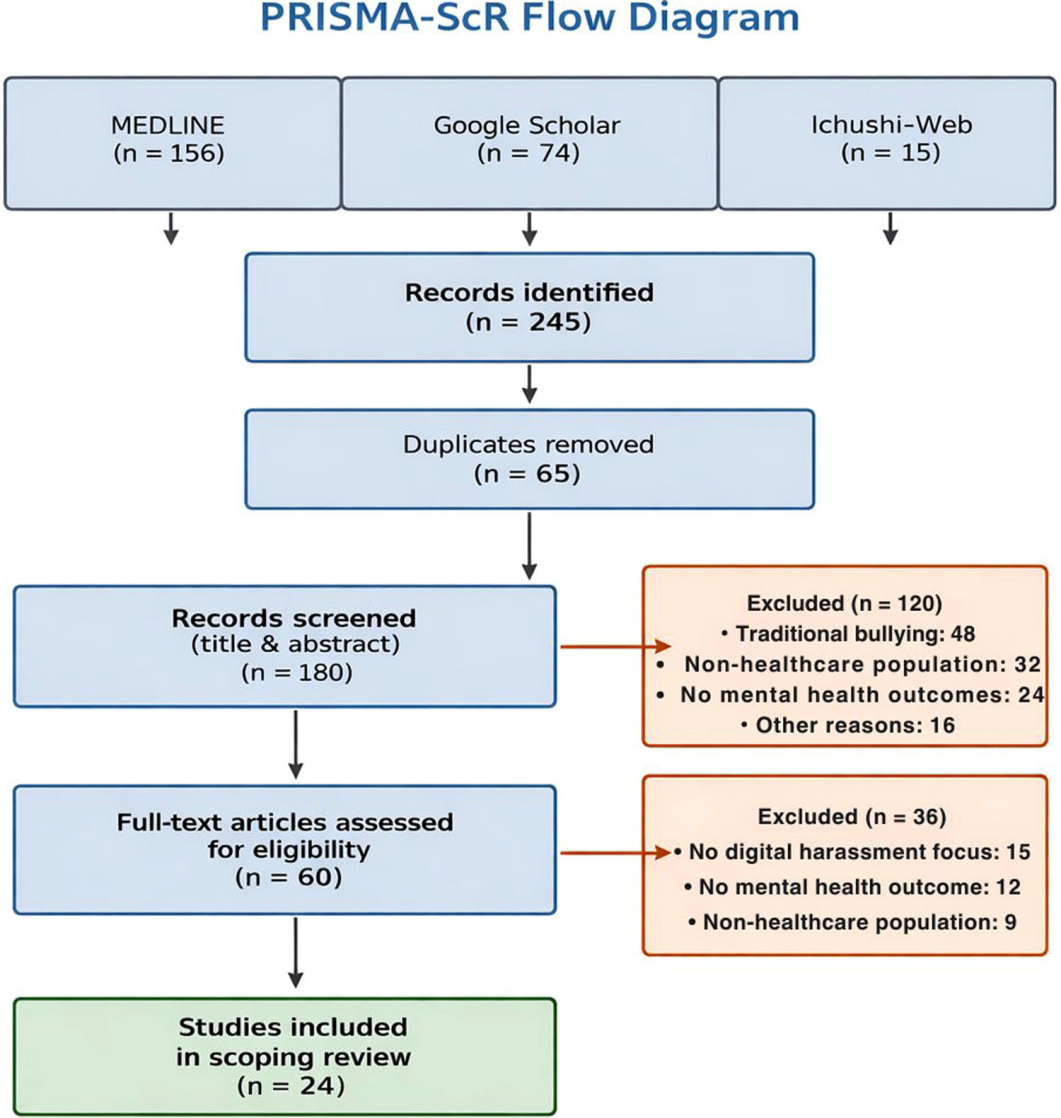
Preferred Reporting Items for Systematic reviews and Meta‐Analyses (PRISMA) flow diagram of the study selection process. Records were identified from MEDLINE (*n* = 156), Google Scholar (*n* = 74), and Ichushi‐Web (*n* = 15), yielding 245 records. After removal of 65 duplicates, 180 records were screened, of which 120 were excluded at title and abstract screening. Sixty full‐text articles were assessed for eligibility, and 36 were excluded. Twenty‐four studies met the inclusion criteria and were included in the scoping review. PRISMA‐ScR, Preferred Reporting Items for Systematic reviews and Meta‐Analyses extension for Scoping Reviews.

Sixty full‐text articles were assessed. Thirty‐six were excluded: 15 lacked a digital harassment focus, 12 did not report mental health outcomes, and 9 examined non‐healthcare populations. Twenty‐four studies met the inclusion criteria (Figure 1).

### Study characteristics

Studies were published between 2012 and 2025, and many were published after 2020. Geographically, studies represented 12 countries: Japan (*n* = 3), the United Kingdom (*n* = 2), the United States (*n* = 2), South Korea (*n* = 2), Italy, Mexico, Australia, Brazil, Canada, China, Malaysia, and Sweden (one each), with seven multinational studies.

Study designs included cross‐sectional surveys (*n* = 14, 58%), systematic reviews (*n* = 4, 17%), one qualitative study, one longitudinal study, one case report, one protocol, and two commentaries. Among cross‐sectional studies that reported sample sizes, participant numbers ranged from 111 to 3684.

Healthcare professional populations varied substantially. Nurses were most studied (*n* = 9), followed by physicians and trainees (*n* = 8), mixed professional samples (*n* = 6), traditional medicine practitioners (*n* = 1), and public health professionals (*n* = 1).

### Types of digital harassment

Workplace cyberbullying was most extensively studied (18/24 studies, 75%), encompassing hostile communications via email or messaging, exclusion from digital communications, rumor spreading, and online incivility from colleagues or supervisors (including representative studies^2^, ^3^, ^13^, ^14^, ^15^, ^16^, ^17^, ^18^).

The UK trainee doctor study reported 46.2% cyberbullying prevalence using broad definitions and a 6‐month recall.^3^ A Korean nurse study found 8% prevalence using restrictive criteria requiring repetition.^13^ A 2025 meta‐analysis synthesizing 21 studies reported victimization rates from 1.5% to 46.6%, with cyberincivility affecting 36.8% of nurses.^15^

Social media harassment and online defamation were examined in seven studies (7/24, 29%), including defamatory reviews on rating websites, coordinated social media campaigns, posting of identifying information without consent, and threatening messages.^1^, ^4^, ^6^, ^7^, ^9^, ^25^, ^26^ The Japanese case report documented severe distress from negative Google Reviews following mandatory child abuse reporting.^4^

Pandemic‐related digital harassment emerged in seven studies (28%) examining COVID‐19, including online abuse related to infection control, stigmatization communicated digitally, harassment related to enforcing public health measures, and misinformation‐based attacks.^6^, ^7^, ^8^, ^9^, ^14^, ^18^, ^25^

Several studies documented overlap among categories, with healthcare professionals experiencing multiple harassment forms simultaneously.

### Mental health outcomes

Studies consistently documented associations between digital harassment and adverse outcomes. Depression (13/24, 54%) and anxiety (12/24, 50%) were the most frequently reported outcomes, with several studies suggesting dose–response relationships for depression. Burnout, assessed in 12 studies (12/24, 50%), showed particularly strong associations with persistent harassment, with some suggesting digital harassment contributes to burnout independent of other stressors.^19^

PTSD symptoms appeared in five studies (5/24, 21%), particularly among those experiencing severe or prolonged harassment. Psychological distress was reported in 16 studies (16/24, 67%). Moral injury appeared in three studies (3/24, 13%), particularly examining harassment following ethically mandated actions.^4^

Work‐related outcomes included job dissatisfaction (11/24 studies, 46%), reduced work engagement (6/24 studies, 25%), and turnover intention (9/24 studies, 38%). Several studies showed that digital harassment predicted turnover intention even after controlling for other workplace stressors. One longitudinal study showed harassment exposure predicted subsequent turnover intention 6 months later.^13^

Physical health outcomes included somatic complaints (three studies), sleep disturbances (five studies), and general health concerns (four studies), possibly reflecting chronic stress from ongoing harassment.

### COVID‐19 pandemic impact

Seven studies examined the pandemic period. A meta‐analysis found 36% of healthcare workers globally experienced stigmatization or violence during the pandemic, representing substantial increases over pre‐pandemic baselines.^8^ A Canadian qualitative study documented threats requiring police involvement, address changes, and security measures.^25^

Risk factors for increased harassment included visible public health roles, enforcement responsibilities, work in COVID‐19 units, and geographic location in high‐conflict communities.

### Geographic distribution and research gaps

Despite an international scope, substantial geographic imbalances characterized the evidence. High‐income English‐speaking countries (the United Kingdom, United States, Canada, and Australia) contributed six country‐specific studies (25%). European countries (Italy, Sweden) contributed 2 (8%). Asian countries contributed 7 (29%), heavily weighted toward South Korea and Japan. Latin American countries (Mexico, Brazil) contributed 2 (8%). The remaining 7 studies (29%) were multinational or global in scope.

Japanese literature was particularly limited: one cross‐sectional COVID‐19 survey,^14^ one study of workplace bullying and patient aggression,^18^ and one case report of harassment following mandatory reporting.^4^ This paucity may reflect systematic underreporting or limited research attention, although firm conclusions cannot be drawn.

Latin American and African contexts were notably underrepresented, with only one Brazilian study and no African studies meeting the inclusion criteria. Middle Eastern countries were similarly absent. This geographic concentration limits generalizability and prevents understanding of how cultural, legal, and organizational contexts shape digital harassment patterns.

## DISCUSSION

This scoping review suggests that digital harassment may represent an emerging occupational challenge for healthcare professionals, with available evidence indicating consistent associations with adverse mental health outcomes across diverse international contexts within the literature identified in this review.^3^, ^13^, ^15^ The synthesis of 24 studies from 12 countries reveals both the apparent international scope of this phenomenon and striking variations in its manifestation, measurement, and impact.

### Prevalence and contextual variation

The wide prevalence range of workplace cyberbullying (1.5%–46.6%) reflects multiple sources of heterogeneity that warrant careful consideration.^3^, ^15^ This heterogeneity reflects differences in study design, outcome measures, and definitions of digital harassment across studies. First, definitional inconsistencies persist across studies. Some researchers operationalize cyberbullying narrowly as repeated hostile digital communications from colleagues, while others employ broader conceptualizations encompassing any negative digital interaction, including single incidents of social media attacks or online defamation. This definitional variation directly impacts prevalence estimates and complicates cross‐study comparisons.

Second, measurement timeframes differ substantially. Studies assessing “ever experienced” cyberbullying yield higher prevalence than those examining experiences within the past 6 months or year, which partly explains the wide range observed across studies.

Third, professional and organizational contexts introduce genuine variation. Trainee doctors in competitive academic medical centers may face different harassment patterns than community‐based nurses, reflecting distinct workplace cultures, hierarchical structures, and digital communication norms.^3^, ^13^ The 46.6% prevalence among UK trainee doctors may reflect both methodological factors and the particularly vulnerable position of trainees within medical hierarchies.

Fourth, cultural and reporting factors introduce additional variation. Cross‐cultural differences in willingness to label negative interactions as harassment may contribute to the observed geographic disparities in prevalence, as discussed in the Japanese literature section below.

### Characteristics of digital harassment reported in included studies

Some included studies described characteristics of online harassment that may differ from offline workplace aggression, including persistence in archived content, broader visibility, and rapid dissemination across platforms.^2^, ^5^ However, these mechanisms were not systematically examined across included studies and should be interpreted cautiously.

Several included studies, particularly a recent Japanese case report^4^ and studies of pandemic‐related harassment,^6^, ^7^ described experiences conceptually related to moral injury, particularly when harassment occurred in response to ethically motivated professional decisions. However, this construct was not consistently measured across included studies and should be interpreted as a hypothesis‐generating observation for future research rather than as a conclusion established by this review.

### COVID‐19 as an inflection point

The COVID‐19 pandemic provided a context in which digital harassment of healthcare professionals was described in several included studies.^6^, ^7^, ^8^, ^9^ Several mechanisms may help explain why pandemic‐related harassment was reported in the included studies.

First, healthcare professionals became visible symbols of pandemic‐related disruptions, restrictions, and mortality.^6^, ^7^, ^8^ Enforcement of infection control measures, delivery of unfavorable COVID‐19 test results, or inability to save critically ill patients has been reported in contexts where healthcare workers became targets for displaced anger and grief.^6^, ^7^, ^9^ Social media platforms have been reported to amplify such scapegoating by providing venues for coordinating harassment campaigns and disseminating misinformation portraying healthcare workers as either incompetent or complicit in supposed pandemic conspiracies.^6^, ^8^, ^9^

Second, pandemic‐related isolation and digital dependency may have increased both the opportunities for harassment and victims' exposure to it. As work and social interactions migrated online, professional boundaries blurred, and healthcare workers spent more time in digital environments where harassment could occur. Simultaneously, social isolation may have reduced access to traditional social support buffers.^7^, ^8^

Third, resource scarcity and ethical dilemmas created morally complex situations ripe for public misunderstanding and criticism. Decisions about resource allocation, admission to intensive care, or end‐of‐life care have been reported as targets for online outrage, particularly when communicated through media coverage or social media rather than through nuanced professional–patient dialogue.^6^, ^7^

The pandemic experience highlights the inadequacy of existing institutional protections. Few healthcare organizations have been reported to have policies addressing social media harassment, protocols for supporting staff targeted by online campaigns, or mechanisms for requesting the removal of defamatory content.^4^, ^6^, ^7^ This systemic unpreparedness may have left individual professionals to navigate harassment largely unsupported.

### Moral injury‐related experiences in included studies

Several included studies described experiences that may be interpreted as moral injury‐related, particularly when digital harassment occurred in response to professional duties, ethically motivated actions, or public‐facing clinical responsibilities.^4^, ^6^, ^7^ However, moral injury was not consistently defined or formally measured across the included studies. Therefore, this finding should be interpreted as hypothesis‐generating rather than conclusive, and future research should examine whether and how digital harassment intersects with moral injury among healthcare professionals.

### Limited literature from Asia within the search scope

Few Japanese‐language or Japan‐based studies were identified within the scope of our English‐ and Japanese‐language search (three studies^4^, ^14^, ^18^). This limited number should be interpreted with caution: it may reflect lower incidence, systematic underreporting, insufficient research attention, or limitations of our search strategy (which did not include Chinese, Korean, or other Asian languages).

Contextual factors that may relate to research output—such as language barriers in publication, differences in research funding priorities, or culturally specific patterns in how workplace harassment is recognized and reported—were not directly assessed in the included studies, and firm conclusions about regional differences cannot be drawn from this review.

This limited representation suggests that additional empirical research from underrepresented regions, including Asia, would help characterize the scope and patterns of digital harassment in diverse healthcare contexts.

### The evidence gap for interventions

Despite increasing attention to digital harassment as an occupational problem, evidence regarding effective interventions remains notably scarce.^22^ The limited intervention literature focuses primarily on traditional face‐to‐face workplace bullying, with minimal evaluation of approaches specifically designed for digital harassment contexts. This evidence gap creates a troubling situation: healthcare professionals face a well‐documented occupational hazard, yet institutions lack evidence‐based guidance for prevention or response.

Several factors may contribute to this intervention evidence gap. Digital harassment's relative novelty may mean insufficient time has elapsed for intervention development and rigorous evaluation. The complex, multifaceted nature of digital harassment—spanning workplace cyberbullying, social media attacks, and online defamation from diverse sources—challenges simple intervention design. The rapidly evolving digital landscape means interventions may become outdated quickly. Additionally, organizational and legal complexities around monitoring digital communications, addressing external harassment sources, and balancing free speech with harassment prevention create implementation challenges.

Priority areas for intervention research include: institutional policies and reporting mechanisms specifically addressing digital harassment; training programs teaching healthcare professionals and administrators to prevent, recognize, and respond to digital harassment; legal and technical approaches for removing or countering defamatory online content; psychological support services addressing the unique features of digital harassment; and organizational culture interventions reducing tolerance for harassment and supporting targets.

### Implications for practice

Several included studies suggested that digital harassment may benefit from being addressed at the organizational and systemic level rather than treated solely as an individual resilience issue.^3^, ^4^, ^25^ Where reported, institutional responses appeared limited, suggesting that policies explicitly addressing digital harassment—including procedures for reporting, evidence documentation, and support of affected staff—may warrant further development and evaluation. The specific design and effectiveness of such institutional responses were not directly evaluated in the included studies and represent an important area for future intervention research.

### Limitations

Several limitations warrant acknowledgment. The heterogeneity in study designs, outcome measures, and harassment definitions precluded quantitative synthesis, limiting our ability to generate precise effect estimates. The predominance of cross‐sectional studies restricts causal inference; while associations between harassment and mental health outcomes are consistent, longitudinal data are needed to establish temporal sequences and distinguish harassment effects from pre‐existing vulnerabilities.

Publication and language bias could have affected findings. Screening was limited to English‐ and Japanese‐language publications, potentially missing relevant studies in other languages, particularly from non‐English‐speaking countries, where digital harassment may be studied using different terminology or published in regional journals. In addition, the MEDLINE search used a focused free‐text keyword strategy without field tags, with a concise mental‐health concept group (mental health, burnout, PTSD, and moral injury); this may have missed studies framed around other outcome terms, such as depression or anxiety, and studies using alternative or emerging terminology. Consistent with the mapping purpose of a scoping review rather than a quantitative synthesis, the review should be read as a map of the available evidence retrieved by this strategy rather than an exhaustive enumeration of all relevant studies. The rapidly evolving digital landscape means that studies published even recently may not capture current harassment patterns, particularly regarding emerging platforms or technologies.

As a scoping review, this study maps the landscape rather than providing a quantitative synthesis of intervention effectiveness. While this approach successfully identified research gaps and characterized the evidence base, systematic reviews with meta‐analyses will be needed to generate precise estimates of prevalence, effect sizes for mental health outcomes, and intervention effectiveness.

Finally, the limited Japanese literature prevents definitive conclusions about harassment prevalence or patterns in Japan. The apparent research gap may reflect reporting bias, different cultural conceptualizations of harassment, or genuine lower prevalence, but current evidence cannot distinguish among these possibilities. Given the exploratory nature of the available evidence, these findings should be interpreted as hypothesis‐generating rather than confirmatory. Furthermore, as a formal critical appraisal of included studies was not conducted in accordance with JBI scoping review methodology, the findings should be interpreted without formal quality weighting.

## CONCLUSIONS

Digital harassment may represent an emerging occupational challenge with potential mental health implications for healthcare professionals. The available evidence suggests associations with depression, anxiety, burnout, moral injury, and workforce attrition, particularly during public health crises such as the COVID‐19 pandemic. This review aimed to map existing evidence rather than provide a quantitative synthesis, and findings should be interpreted as an overview of the current literature landscape within our English‐ and Japanese‐language search scope. Addressing this challenge may benefit from institutional policies, legal frameworks, and tailored psychological support, although the specific design and effectiveness of such measures were not directly evaluated in the included studies and represent priorities for future intervention research.

Future research priorities include longitudinal studies establishing temporal relationships and long‐term consequences, intervention trials evaluating prevention strategies and support services, cross‐cultural investigations exploring harassment patterns and protective factors across diverse contexts, studies examining the experiences of professionals in underrepresented regions such as Japan, and research developing and validating digital harassment measurement instruments capturing the phenomenon's unique dimensions.

Healthcare professionals perform essential societal functions, often under challenging conditions requiring difficult ethical decisions. These findings suggest that institutional, legal, and professional protections may be warranted to enable healthcare workers to fulfill these functions without facing online vilification and psychological harm. Further research and policy attention to digital harassment in healthcare settings appear important for sustaining a healthy healthcare workforce.

## AUTHOR CONTRIBUTIONS

Kyohei Otani conceived and designed the study, conducted the literature search, synthesized data, and drafted the manuscript. Tomohiro Kinoshita and Ryota Shindo contributed to data collection and study selection. Shogo Kurushima reviewed extracted data and methodological characteristics. All authors approved the final manuscript.

## CONFLICT OF INTEREST STATEMENT

The authors declare no conflicts of interest.

## ETHICS APPROVAL STATEMENT

Ethics approval was not required because this study was based exclusively on published literature.

## PATIENT CONSENT STATEMENT

Not applicable. This study did not involve identifiable patient data.

## CLINICAL TRIAL REGISTRATION

Not applicable.

## Supporting information

Supporting File 1

## Data Availability

All data analyzed in this study are derived from publicly available published literature. The extracted data supporting the findings of this study are available from the corresponding author upon reasonable request.
