# Supplementary material for: Digital harassment and its mental health impact among healthcare professionals: A scoping review
Source: PCN Rep. 2026 Jun 11;5(2):e70362. doi: 10.1002/pcn5.70362 (PMC13254817; doi:10.1002/pcn5.70362)
Supplement: Supplementary file 1 — Supporting File 1 [file PCN5-5-e70362-s001.docx]

**Supplementary Material**

**Supplementary Table S1. MEDLINE (PubMed) Electronic Search Strategy**

*Database: MEDLINE via PubMed | Search date: December 31, 2025 | Date range: Inception to December 31, 2025*

| **Concept Group** | **Search Terms** | | | |
| --- | --- | --- | --- | --- |
| **Concept 1 Healthcare professionals** | healthcare worker OR physician OR doctor OR nurse OR medical professional OR healthcare professional |  |  |  |
| **Concept 2 Digital harassment** | cyberbullying OR digital harassment OR online harassment OR online defamation OR social media attack |  |  |  |
| **Concept 3 Mental health outcomes** | mental health OR burnout OR PTSD OR moral injury |  |  |  |
| **Combined** | **(Concept 1) AND (Concept 2) AND (Concept 3)** |  |  |  |
| **Filters** | Screening was limited to English and Japanese publications \| Date: Inception to December 31, 2025 \| Database: MEDLINE via PubMed |  |  |  |
| **Results** | The original MEDLINE/PubMed search conducted on December 31, 2025 identified 156 records, which formed the basis of the screening process. When the clarified search strategy shown above was rerun with the same end date, it retrieved 154 records. This difference did not affect the screening process, PRISMA flow, included studies, or conclusions of the review. |  |  |  |

*Note: The same three-concept search strategy was applied to Google Scholar (search date: December 31, 2025), yielding approximately 17,800 results. In accordance with established scoping review practice for this non-indexed database, the first 200 relevance-sorted results were screened, of which 74 were identified as potentially relevant. Ichushi-Web was searched using Japanese MeSH equivalents: デジタルハラスメント, サイバーハラスメント, ネットハラスメント, 医療従事者, メンタルヘルス.*

**Supplementary Table S2. PRISMA Extension for Scoping Reviews (PRISMA-ScR) Checklist**

*Based on: Tricco AC, Lillie E, Zarin W, et al. PRISMA Extension for Scoping Reviews (PRISMA-ScR). Ann Intern Med. 2018;169(7):467–473.*

| **Section/Topic** | **#** | **PRISMA-ScR Checklist Item** | **Location in Manuscript** |
| --- | --- | --- | --- |
| **TITLE** | **1** | Identify the report as a scoping review. | Title page |
| **ABSTRACT** | **2** | Provide a structured summary including background, objectives, eligibility criteria, sources of evidence, charting methods, results, and conclusions. | Abstract (structured: Background, Objective, Methods, Results, Conclusions) |
| **INTRODUCTION Rationale** | **3** | Describe the rationale for the review in the context of what is already known. | Introduction, paragraphs 1–5 |
| **INTRODUCTION Objectives** | **4** | Provide an explicit statement of the questions and objectives with reference to key elements (population, concept, context). | Introduction, final paragraph: five enumerated objectives (1)–(5) |
| **METHODS Protocol & Registration** | **5** | Indicate if a review protocol exists and if it was registered. | Methods: "This scoping review was not prospectively registered." |
| **METHODS Eligibility Criteria** | **6** | Specify characteristics used as eligibility criteria and provide a rationale. | Methods, Eligibility Criteria: four inclusion and four exclusion criteria |
| **METHODS Information Sources** | **7** | Describe all information sources and date of most recent search. | Methods: MEDLINE, Google Scholar, Ichushi-Web; search date December 31, 2025 |
| **METHODS Search** | **8** | Present the full electronic search strategy for at least one database. | Supplementary Table S1: full MEDLINE search strategy |
| **METHODS Selection** | **9** | State the process for selecting sources of evidence. | Methods: two-reviewer independent screening, third reviewer for disagreements |
| **METHODS Data Charting** | **10** | Describe the methods of charting data. | Methods: standardized extraction form pilot-tested on five studies |
| **METHODS Data Items** | **11** | List and define all variables for which data were sought. | Methods: author/year, design, country, population, harassment type, outcomes, instruments, key findings, limitations |
| **METHODS Critical Appraisal** | **12** | If done, provide a rationale for conducting a critical appraisal. | Not applicable; per JBI scoping review methodology, formal critical appraisal of included studies was not conducted (see Methods). Methodological characteristics are described narratively in the Results and Limitations. |
| **METHODS Synthesis** | **13** | Describe the methods for handling and summarising data. | Methods: narrative synthesis around four domains; no meta-analysis |
| **RESULTS Selection** | **14** | Give numbers of sources screened, assessed, and included, with reasons for exclusions. | Results: PRISMA flow (Figure 1) |
| **RESULTS Characteristics** | **15** | Present characteristics for which data were charted and provide citations. | Results; Table 1 (all 24 studies) |
| **RESULTS Critical Appraisal** | **16** | If done, present data on critical appraisal. | Not applicable; formal critical appraisal was not conducted (see Item 12). |
| **RESULTS Individual Sources** | **17** | For each source, present the relevant data charted. | Table 1: individual study data |
| **RESULTS Synthesis** | **18** | Summarise and/or present charting results as they relate to objectives. | Results: Types of Digital Harassment, Mental Health Outcomes, COVID-19 Impact, Geographic Distribution |
| **DISCUSSION Summary** | **19** | Summarise the main results and consider relevance to key stakeholders. | Discussion: all subsections address objectives 1–5 |
| **DISCUSSION Limitations** | **20** | Discuss limitations of the scoping review process. | Discussion, Limitations: heterogeneity, cross-sectional predominance, publication/language/Google Scholar bias |
| **DISCUSSION Conclusions** | **21** | Provide a general interpretation with implications for future research and practice. | Discussion, Conclusions; Future Research Priorities |
| **FUNDING** | **22** | Describe sources of funding. | Funding: "No external funding was received." |

*All 22 items of the PRISMA-ScR are addressed in this manuscript. This scoping review was not prospectively registered (Item 5). In line with JBI scoping review methodology, formal critical appraisal of included studies was not conducted; consequently, Items 12 and 16 are marked as Not applicable above. Methodological characteristics of included studies are described narratively in the Results and acknowledged in the Limitations.*
